# Prediction model of gleason score upgrading after radical prostatectomy based on a bayesian network

**DOI:** 10.1186/s12894-023-01330-6

**Published:** 2023-10-07

**Authors:** Guipeng Wang, Xinning Wang, Haotian Du, Yaozhong Wang, Liguo Sun, Mingxin Zhang, Shengxian Li, Yuefeng Jia, Xuecheng Yang

**Affiliations:** 1 Department of Urology, The Affiliated Hospital of Qingdao University, Qingdao, China; 2 Department of Urology, JuXian People’s Hospital, Rizhao, China

**Keywords:** Prostate cancer, Prostate needle biopsy, Bayesian network, Prediction model

## Abstract

**Objective:**

To explore the clinical value of the Gleason score upgrading (GSU) prediction model after radical prostatectomy (RP) based on a Bayesian network.

**Methods:**

The data of 356 patients who underwent prostate biopsy and RP in our hospital from January 2018 to May 2021 were retrospectively analysed. Fourteen risk factors, including age, body mass index (BMI), total prostate-specific antigen (tPSA), prostate volume, total prostate-specific antigen density (PSAD), the number and proportion of positive biopsy cores, PI-RADS score, clinical stage and postoperative pathological characteristics, were included in the analysis. Data were used to establish a prediction model for Gleason score elevation based on the tree augmented naive (TAN) Bayesian algorithm. Moreover, the Bayesia Lab validation function was used to calculate the importance of polymorphic Birnbaum according to the results of the posterior analysis and to obtain the importance of each risk factor.

**Results:**

In the overall cohort, 110 patients (30.89%) had GSU. Based on all of the risk factors that were included in this study, the AUC of the model was 81.06%, and the accuracy was 76.64%. The importance ranking results showed that lymphatic metastasis, the number of positive biopsy cores, ISUP stage and PI-RADS score were the top four influencing factors for GSU after RP.

**Conclusions:**

The prediction model of GSU after RP based on a Bayesian network has high accuracy and can more accurately evaluate the Gleason score of prostate biopsy specimens and guide treatment decisions.

**Supplementary Information:**

The online version contains supplementary material available at 10.1186/s12894-023-01330-6.

## Introduction

Prostate cancer is one of the most common malignant tumours of the genitourinary system in elderly men. Its incidence ranks second in the global male cancer incidence spectrum, and its incidence in China is increasing yearly [1, 2]. Prostate biopsy is the gold standard for the diagnosis of prostate cancer. The Gleason score of biopsy is an important factor for clinicians to assess the biological behaviour of tumours and one of the important bases for selecting treatment options before radical prostatectomy (RP) [3]. However, the Gleason score of prostate biopsy is still inconsistent with that of radical prostatectomy. The overall accuracy of Gleason grade of prostate biopsy was reported to be only 63%, and approximately 30% of patients had an upgrade of the score [4]. This may lead clinicians to underestimate the risk of disease, affecting patient prognosis. Therefore, the establishment of an accurate prediction model for Gleason score elevation is of great guiding importance for the assessment of tumour risk and the formulation of treatment plans for prostate cancer patients [5].

Bayesian theory is a statistical theory corresponding to classical statistics that introduces prior information on the basis of sample information and synthetically investigates the two aspects of information to make inferences about the population. The structure of the Bayesian network is a directed acyclic graph, which can present the joint probability density between high-dimensional variables. The TAN Bayesian network (Tree-Augmented Nave Bayesian network) is an extension of the classical Bayesian network model that can address correlated variables and has good predictive ability for high-dimensional data. Bayesian networks have been widely used in medicine, such as in survival models, infectious disease models, decision analysis and gene network analysis [6–8]. Therefore, we applied the Bayesian network to establish the prediction model of increases in Gleason scores and combined it with significance theory while also calculating the weight of each influencing factor before surgery and discussing its clinical guiding importance.

## Materials and methods

### Patients and data collection

A total of 573 patients with prostate cancer underwent radical prostatectomy (RP) in our centre from January 2018 to May 2021.

The inclusion criteria were as follows: (1) both prostate biopsy and RP were performed in our centre; (2) the interval between biopsy and RP was less than 60 days; and (3) detailed clinical and pathological data were available.

The exclusion criteria were as follows: (1) patients who had a history of radiotherapy and endocrine therapy before RP; and (2) patients who had a history of prostate surgery before RP.

A total of 356 patients were included in this study.

### Methods

All patients underwent transperineal standard systematic 12-core biopsy and cognitive MRI/US fusion targeted biopsy. A minimum of two cores were taken for each targeted lesion, followed by a standard 12-core biopsy. The biopsy was performed by senior urologists who had passed the learning curve of the procedure, and the examinations and diagnoses of postoperative pathological specimens were completed by two pathologists with senior professional titles. The Gleason score was scored according to the 2014 International Society of Urological Pathology (ISUP) consensus conference on Gleason grading of prostate cancer. The mpMRI protocol followed the Prostate Imaging Reporting & Data System (PI-RADS) guidelines with T2-weighted, diffusion-weighted, and dynamic contrast-enhanced sequences. The PI-RADS score was assigned by senior radiologists with subspecialist experience in prostate MRI.

We defined the GSU as follows: (1) the total GS score of the specimen after RP was greater than that of the biopsy specimen; and (2) the Gleason score changed from 3 + 4 at biopsy to 4 + 3 after RP.

### Inclusion factors and pretreatment

We analysed clinical data, including age, body mass index (BMI), total prostate-specific antigen (tPSA), prostate volume, total prostate-specific antigen density (PSAD), clinical stage, pathological characteristics of the biopsy specimen, PI-RADS score and pathological characteristics after RP. The abovementioned indicators were selected with reference to a previous study on the analysis of risk factors for GSU [9–12]. All of the continuous variables were transformed to discrete variables for the BN analysis and are expressed as frequencies and percentages. Categorical variables are presented as frequencies and percentages.

### Bayesian network analysis method

To evaluate the performance of the model more accurately, a stratified sampling strategy was used to split the dataset into a training dataset and a test dataset. 70% (70%, 249 cases) of the patients were used as the training dataset to establish the model by using the tree-augmented naive Bayes (TAN) algorithm, and the remaining 30% (107 cases) of the patients were used as the test dataset to test the model. The reliability and precision in the confusion matrix were expressed as percentages, and the receiver operating characteristic curve (ROC curve) was plotted by locking the target. All of the abovementioned variables were included, and Bayesia Lab software was used to establish a prediction model based on the TAN algorithm. The confusion matrix, ROC curve and area under the curve (AUC) were used to evaluate the quality of the model. A larger confusion matrix corresponded to a higher accuracy of the model. Moreover, a larger AUC value corresponded to a higher accuracy of the model. After evaluating the accuracy of the model, the Bayesia Lab software was used to perform a priori analysis of 14 variables and a posterior analysis with GSU as the target variable and the remaining factors as the attribute variables. The results of the posterior analysis combined with the polymorphic Birnbaum importance calculation were used to calculate the importance ranking of the attribute variables.

## Results

**Fig. 1:**
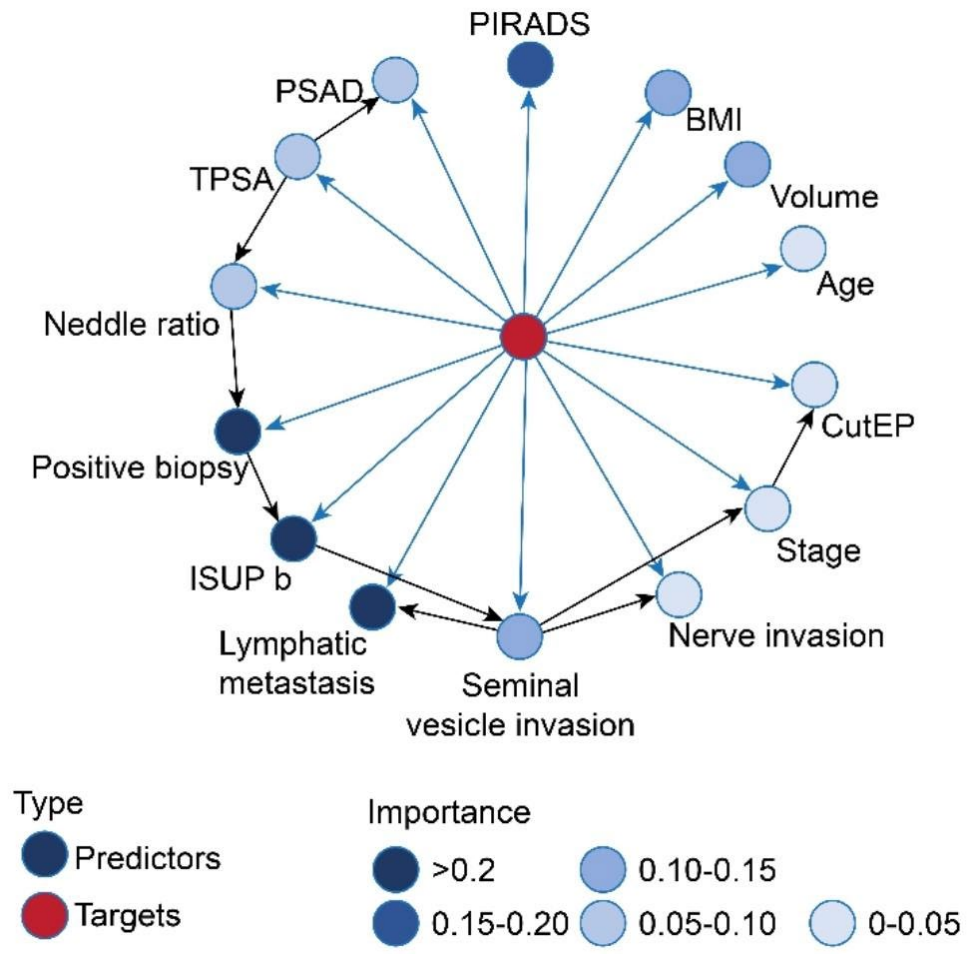
Bayesian prediction model based on 14 clinical predictors

**Fig. 2:**
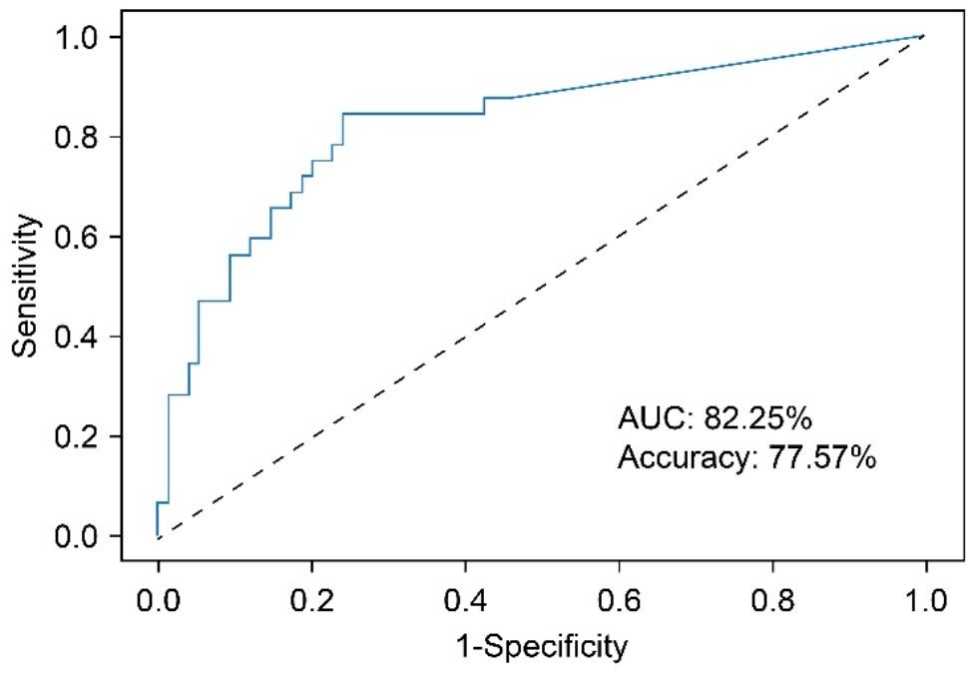
Receiver operating curve based on 14 clinical predictors

A total of 110 patients (30.89%) had Gleason score upgrades. All of the factors in Table 1 were incorporated to establish the TAN Bayesian network model via the Bayesia Lab software. The obtained model demonstrated the relationship between the 14 factors and GSU and the relationship between the 14 factors (Fig. 1). Red nodes represent the target variable GSU, blue nodes represent the attribute variable, and the darker colour indicates a more important means of predicting the GSU. According to the ROC curve established by the data of the model validation set (Fig. 2), the AUC of the model was 82.25%.

**Table 1:** Pretreatment of GSU risk factors

Factor	Value	No GSU, N (%)	GSU, N (%)
Age	0(<60)	26 (7.30)	17(4.78)
	1(60–70)	142(39.88)	60(16.85)
	2(>70)	78(21.91)	33(9.27)
BMI	0(<18 kg/m²)	4(1.12)	3(0.84)
	1(18–24 kg/m²)	85(23.88)	42(11.79)
	2(>24 kg/m²)	157(44.10)	65(18.26)
PSA	0(<10 µg/L)	41(11.52)	27(7.58)
	1(10–20 µg/L)	70(19.66)	28(7.87)
	2(>20 µg/L)	135(37.92)	55(15.44)
Positive cores	0(<3)	76(21.34)	60(16.85)
	1(≥3)	170(47.75)	50(14.04)
% of positive cores	0(<50%)	116(32.58)	60(16.85)
	1(≥50%)	130(36.52)	50(14.04)
Prostate volume	0(<40 ml)	98(27.53)	69(19.38)
	1(40–60 ml)	81(22.75)	25(7.02)
	2(>60 ml)	67(18.82)	16(4.49)
PSAD	0(<0.25)	50(14.04)	32(8.99)
	1(0.25–0.5)	59(16.57)	22(6.18)
	2(0.5-1)	55(15.45)	23(6.46)
	3(>1)	82(23.03)	33(9.27)
PI-RADS	3	41(11.52)	6(16.85)
	4	140(39.33)	63(17.70)
	5	65(18.26)	41(11.52)
Clinical stage	<T3	162(45.50)	61(17.13)
	≥T3	84(23.60)	49(13.76)
Lymphatic metastasis	yes	23(65.71)	2(0.56)
	no	223(62.64)	108(30.33)
PSM	yes	106(29.78)	51(14.33)
	no	140(39.33)	59(16.57)
SVI	Yes	66(18.54)	18(5.06)
	no	180(50.56)	92(25.84)
NI	yes	143(40.17)	69(19.38)
	no	83(23.31)	41(11.52)
ISUP grading	1	30(8.43)	48(13.48)
	2	19(5.34)	13(3.65)
	3	36(10.11)	13(3.65)
	4	42(11.80)	32(8.99)
	5	119(33.43)	4(1.12)

The confusion matrix is shown in Table 2. The number of correct predictive values included 19 GSU cases and 64 cases without G score upgrades. The number of false predictive values included 11 GSU cases and 13 cases with Gleason scores not upgraded. The overall accuracy of the confusion matrix was 77.57%, the sensitivity was 59.8%, and the specificity was 85.33%.

**Table 2:** GSU prediction model test set confusion matrix

	n	N		Reliability		Accuracy	
		No GSU 75	GSU 32	No GSU	GSU	No GSU	GSU
No GSU	77	64	13	83.12%	16.88%	85.33%	40.62%
**GSU**	**30**	**11**	**19**	**36.67%**	**63.33%**	**14.67%**	**59.38%**

**Fig. 3:**
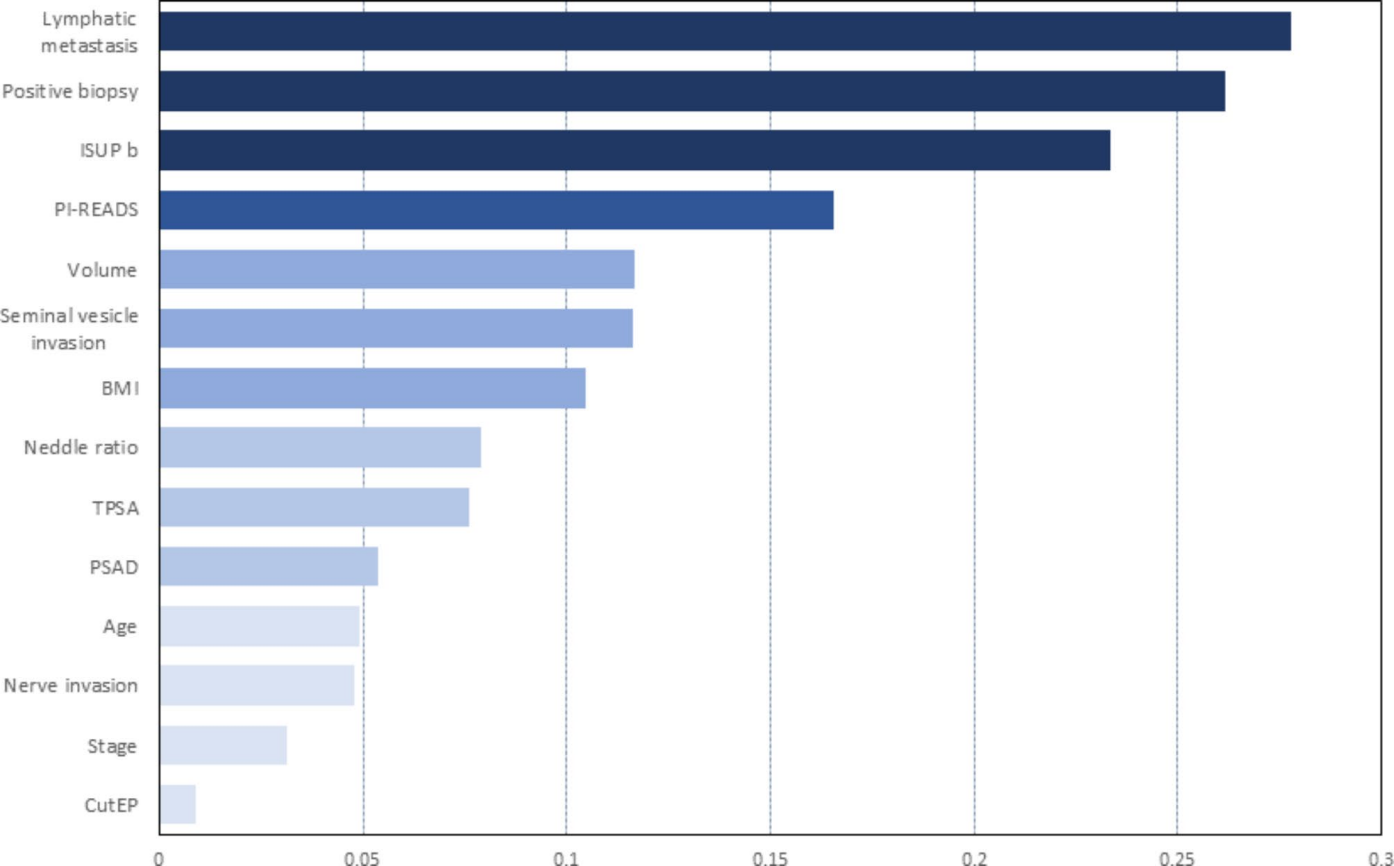
Ranking of importance of 14 clinical predictors

Based on the Bayesian network model, the Bayesia Lab analysis verification function was used to perform prior probability statistics, posterior analysis, importance calculation and ranking of the influencing factors of the GSU (Table 3). The results of importance ranking (Fig. 3) showed that lymph node metastasis (0.2777), number of positive puncture needles (0.2617) and ISUP grade (0.2334) were in the first importance interval. Moreover, PI-RADS score (0.1654), prostate volume (0.1168), seminal vesicle invasion (0.1164) and BMI (0.1046) were in the second importance range.

**Table 3:** Analysis of GSU-related factors after RP to verify the results

Factor	Priori probability	Posterior probability	Importance	Rank
Age	0.1446	0.6111	0.0492	11
	0.5542	0.7029		
	0.3012	0.6933		
BMI	0.0241	0.5000	0.1046	7
	0.2972	0.6757		
	0.6787	0.6982		
PSA	0.1847	0.587	0.0762	9
	0.2771	0.7246		
	0.5382	0.7015		
Positive biopsy	0.3695	0.5217	0.2617	2
	0.6305	0.7834		
Needle ratio	0.5141	0.6484	0.0789	8
	0.4859	0.7273		
Prostate volume	0.4779	0.605	0.1168	5
	0.2932	0.7534		
	0.2289	0.7719		
PSAD	0.2289	0.5965	0.0539	10
	0.2088	0.7115		
	0.2209	0.6909		
	0.3414	0.7294		
PI-RADS	0.1124	0.8929	0.1654	4
	0.5823	0.7034		
	0.3052	0.5789		
Clinical stage	0.6386	0.6981	0.0314	13
	0.3614	0.6667		
PSM	0.5582	0.6906	0.0088	14
	0.4418	0.6818		
LM	0.9277	0.6667	0.2777	1
	0.0723	0.9444		
SVI	0.7510	0.6578	0.1164	6
	0.2490	0.7742		
IV	0.3253	0.6543	0.0481	12
	0.6747	0.7024		
ISUP grading	0.2008	0.32	0.2334	3
	0.1044	0.5769		
	0.1365	0.7647		
	0.2289	0.5965		
	0.3293	0.9756		

## Discussion

The Gleason score that was obtained via prostate biopsy is an important basis for evaluating cancer risk before surgery and making treatment plans. When considering patients who choose active surveillance, the pathological information obtained by biopsy is an important method to assist clinical decision-making [13]. Needle biopsy technology is constantly improving, but inconsistencies in the Gleason score between biopsy and surgical specimens are frequently reported. GSU can lead clinicians to underestimate the risk of tumours, which results in a poor prognosis of tumours, including positive surgical margins and biochemical recurrence [14]. This subsequently affects the accuracy of disease diagnosis and treatment and the survival time and quality of life of patients while also increasing the economic and mental burdens of patients. Therefore, we require a specific predictive model to evaluate the risk of Gleason upgrade in patients before surgery to better evaluate the risks of patients and guide clinical decision-making.

In recent years, prediction models based on big data and artificial intelligence have become a hot spot in clinical research. Previous prediction models for GSU are mostly constructed by using nomogram models; however, due to different risk factors included in different studies, the AUC and accuracy of the prediction models can vary considerably. The accuracy of the nomogram model constructed by Wang [15] based on PSA, biopsy Gleason score, postoperative Gleason score and clinical staging was 78.9%. Moreover, Chun’s model [16] was externally verified by their research data, and the model was considered to be inaccurate.

In addition, with the progress of clinical research in recent years, an increasing number of factors have been confirmed to be related to GSU, such as the ratio of positive puncture cores, PSAD and PI-RADS score. The nomogram model only includes independent predictors, and when the nomogram model contains too many predictors, it is easy to fit, which is likely to lead to the failure of the prediction model.

Bayesian networks are an effective tool that combines probability theory and graph theory to address uncertainty reasoning and data analysis. It can analyse the problem structure according to the principle of probability theory to reduce the complexity of reasoning and calculation. Moreover, the TAN Bayesian network is an extension of the classical Bayesian network model that can address correlated variables and has good prediction ability for high-dimensional data [17]. In recent years, there have been more studies using TAN Bayesian networks to construct clinical models, and the constructed models have better prediction performance [18–20]. Moreover, the Bayesian network model is not limited to independent prognostic factors and can accept nonlinear data, thus making full use of all of the variable information; additionally, it can more comprehensively predict the outcome. When the amount of data is large enough, the incorporation of as many factors as possible is expected to result in a prediction model that is closer to real-world scenarios.

We included 14 relevant variables to construct the TAN Bayesian model, thus resulting in an AUC of 82.25%. Moreover, the confusion matrix analysis showed that the accuracy of the Bayesian prediction model was 77.57%, which has a good prediction effect. This reflects the fact that after more variables are included, the accuracy and prediction performance of the Bayesian model is better than that of the previous nomogram model, enabling improved evaluation of the influence of many preoperative variables on Gleason upgrading. Based on this model, we applied importance theory to calculate the importance ranking of GSU risk factors.

The results showed that lymph node metastasis, number of positive puncture needles, ISUP grade and PI-RADS score were the top four predictors of GSU. These results suggest that patients with suspected lymph node metastasis on preoperative imaging are at higher risk of GSU. Prostate cancer with lymph node metastasis may be highly malignant, and tumour tissue with high G may be missed at biopsy. In addition, the number of positive needles is often related to the tumour volume, and studies have shown that an adequate number of needles can improve the consistency of the score and reduce the risk of increasing the score [21]. For patients with large prostate cancer, an appropriate increase in the number of biopsy cores can improve the scoring consistency and avoid missing high-grade cancer. The PI-RADS score has important value in the evaluation of prostate cancer. It has been reported that a lower PI-RADS score corresponds to a lower Gleason score. Furthermore, a PI-RADS score of 4–5 is an independent risk factor for GSU, and a higher PI-RADS score corresponds to a higher incidence of GSU [22].

In addition, the TAN Bayesian network model can depict the conditional dependence network between the dependent variable and the predictor variable and display it in the form of a dendrogram, which is simple and intuitive. Our model showed that seminal vesicle invasion was associated with lymph node metastasis and nerve invasion, which was consistent with the clinical characteristics of advanced patients. Clinical stage was associated with positive surgical margins, which is consistent with previous studies in which more advanced tumours had a higher incidence of positive surgical margins [23]. This suggests that we should pay attention to the prevention of positive margins when RP is performed for patients with advanced stages. The presentation of the dendrogram enables us to understand the mechanism of GSU from a broader perspective and to identify the interaction between various factors.

This study is a preliminary attempt to apply Bayesian networks in the field of prostate cancer; however, there were still some limitations. First, due to the limitations of this study being a single-centre study and the lack of external validation, the predictive effect of the model on different populations is still unclear. In addition, Sheridan [24] found that the risk of progression of prostate cancer was only 3% when it was diagnosed within 1 year; however, some scholars believe that an interval that is too long will increase the risk of score increases in low-risk patients [25]. Patients with an interval of less than 60 days between puncture and RP were included in this study to reduce the impact of tumour progression on score escalation. Future prospective multicentre studies based on large populations are expected to further optimize the GSU model to make the risk stratification of patients more accurate and personalized, thus ultimately achieving the purpose of improving the prognoses of patients and improving their quality of life.

## Conclusion

The predictive model of Gleason score upgrade after radical prostatectomy based on the Bayesian network has high predictive power, which is better than that of the previous nomogram model. This study can be used to guide clinicians to evaluate the risk of GSU, obtain a more accurate Gleason score before surgery and select the most appropriate treatment plan for patients.

### Electronic supplementary material

Below is the link to the electronic supplementary material.

Supplementary Material 1

## Data Availability

The datasets used and analysed during the current study are available from the corresponding author on reasonable request.
